# Prognostic Value of Blood Urea Nitrogen for Acute Kidney Injury and Mortality in Vasculitis: A Large Cohort Study Using Multivariate Joint Model and Machine Learning

**DOI:** 10.3390/diagnostics16050665

**Published:** 2026-02-25

**Authors:** Si Chen, Rongfeng Liu, Yongzhi Zhang, Yan Wang, Haixia Luan, Xiaoli Zeng, Hui Yuan

**Affiliations:** Department of Clinical Laboratory, Beijing Anzhen Hospital, Capital Medical University, Anzhen Road No. 2, Chaoyang District, Beijing 100029, China; chensi888.hi@163.com (S.C.); aser1981@sina.com (R.L.); zhyzh0@163.com (Y.Z.); 13801041202@163.com (Y.W.); haixialuan888@163.com (H.L.)

**Keywords:** vsculitis, acute kidney injury, blood urea nitrogen, mortality, machine learning

## Abstract

**Background:** Acute kidney injury (AKI) is a serious complication in vasculitis patients and may adversely affect prognosis. However, the role of blood urea nitrogen (BUN) as a predictor of AKI and mortality in vasculitis has not been fully elucidated. **Methods:** We retrospectively analyzed 701 patients with large-, medium-, and small-vessel vasculitis from the MIMIC-III/IV databases to evaluate the relationship between BUN, AKI occurrence, and mortality. AKI was defined according to the KDIGO serum creatinine criteria. Logistic and Cox regression models, restricted cubic spline (RCS) analyses, and multiple machine learning models were employed to identify risk factors and assess predictive performance. **Results:** AKI occurred in 25.1% (176/701) of vasculitis patients and was associated with significantly higher 30- and 365-day mortality rates (*p* < 0.05). Multivariable logistic regression identified BUN as an independent predictor of AKI (OR: 1.03; 95% CI: 1.02–1.05; *p* < 0.0001). Patients in the highest BUN tertile had a 5.67-fold greater risk of AKI compared to the lowest tertile (*p* < 0.0001). The Cox regression confirmed BUN as an independent predictor of 30- and 365-day mortality among patients with AKI (*p* < 0.05). The RCS analysis identified a critical BUN threshold of 32 mg/dL, above which the mortality risk markedly increased. Machine learning models further validated the prognostic significance of BUN and age, with the logistic regression model achieving the highest predictive accuracy (area under the curve: 0.904). **Conclusions:** BUN is a practical predictor of AKI and mortality in vasculitis and may assist early risk stratification in this population.

## 1. Introduction

Vasculitis refers to a heterogeneous group of disorders characterized by inflammation of the vessel wall, which may result in ischemia, necrosis, or aneurysmal changes. The 2012 revised International Chapel Hill Consensus Conference established a standardized nomenclature system for vasculitides, primarily classifying them based on the size of the affected vessels (large, medium, or small) [1]. Vasculitis frequently involves the kidneys due to their extensive vascular network, making renal injury a common and sometimes initial manifestation of these disorders. Renal involvement can manifest as glomerulonephritis, vascular occlusion, and ultimately acute kidney injury (AKI) through diverse pathophysiological mechanisms [2,3]. Small-vessel vasculitides, particularly those associated with antineutrophil cytoplasmic antibodies (ANCA)—such as granulomatosis with polyangiitis (GPA), microscopic polyangiitis (MPA), and eosinophilic granulomatosis with polyangiitis—are the most frequent causes of renal vasculitis. These entities characteristically produce pauci-immune necrotizing crescentic glomerulonephritis, leading to rapidly progressive glomerulonephritis (RPGN) and AKI if left untreated [2,4,5,6]. Medium-vessel vasculitis, exemplified by polyarteritis nodosa (PAN), primarily affects medium-sized renal arteries, causing ischemia, infarction, and, in severe cases, hemorrhage due to aneurysm rupture [2,7]. Large-vessel vasculitides, such as giant cell arteritis (GCA), rarely involve renal parenchyma directly but can cause secondary ischemic injury through renal artery stenosis or occlusion, occasionally leading to renovascular hypertension and AKI [3,8]. Although Behçet’s disease (BD) is primarily a multisystem vasculitis affecting mucocutaneous, ocular, and vascular systems, renal involvement—though rare—is increasingly recognized [9,10,11]. Renal disease in BD is often mild and asymptomatic, highlighting the need for routine renal function monitoring for early detection [9]. Early recognition and treatment of vasculitic renal involvement are critical for preserving renal function and improving patient outcomes [4,6,10].

While creatinine (Cr) is the most widely used marker of renal dysfunction, its elevation is often delayed in acute settings and is influenced by muscle mass, age, sex, and chronic comorbidities [12,13,14]. Prior studies have demonstrated limitations of Cr as a filtration marker, particularly in glomerular disease and sepsis, where reduced Cr production further impairs its reliability [14,15]. In contrast, blood urea nitrogen (BUN) rises earlier in response to renal hypoperfusion, catabolic stress, and systemic inflammation—mechanisms frequently observed in vasculitis [16]. Recent evidence further supports the prognostic relevance of BUN in acute and critical illness settings, where elevated BUN has been associated with increased mortality and adverse renal outcomes [17]. Biochemically, BUN reflects both renal clearance and protein catabolism, providing a more dynamic indicator of acute physiological stress [18]. However, BUN levels are also influenced by fluid status, protein intake, gastrointestinal bleeding, and catabolic state [19]. Therefore, elevated BUN may reflect a combination of renal dysfunction and systemic metabolic stress rather than isolated impairment of glomerular filtration [20]. This multifactorial nature may partly explain its strong association with adverse outcomes in critically ill vasculitis patients [21]. Previous studies have suggested the prognostic significance of elevated BUN in vasculitis subtypes with renal involvement, including BD, GPA, and PAN [22,23,24]; however, BUN has not been specifically evaluated as a predictor of AKI or mortality across heterogeneous vasculitis populations. Given its physiological sensitivity to hemodynamic instability and inflammatory burden, BUN may serve as a more responsive indicator of acute renal stress than Cr in vasculitis.

Traditional regression models may be limited in capturing complex nonlinear relationships and interactions among inflammatory, renal, and hemodynamic parameters. Machine learning approaches offer advantages by modeling higher-order interactions and improving predictive performance in heterogeneous clinical datasets. Machine learning algorithms have demonstrated superior accuracy in AKI prediction across large electronic health record datasets and continuous real-time risk forecasting in critical care settings [25,26]. To date, no study has applied machine learning to evaluate BUN as a predictor of AKI and mortality in vasculitis.

Therefore, this study aims to investigate the incidence and risk factors of AKI in a large cohort of patients with vasculitis and to further explore the risk factors associated with mortality in vasculitis patients experiencing AKI complications.

## 2. Methods

### 2.1. Data Source

This study was conducted using data from two publicly available critical care databases: Medical Information Mart for Intensive Care (MIMIC)-III (version 1.4) [27] and MIMIC-IV (version 3.1) [28]. Both were developed by the Beth Israel Deaconess Medical Center in collaboration with the MIT Laboratory for Computational Physiology. The MIMIC-III database includes clinical data from adult ICU patients admitted between 2001 and 2008 [29], while MIMIC-IV extends this timeframe by incorporating records from ICU admissions between 2008 and 2019 [30]. Thus, this study retrospectively assessed patient data from BIDMC covering the years 2001–2019. The first author (S.C.) was granted access to these databases after completing the Collaborative Institutional Training Initiative course on human subject protection (certification number: 64388854). As all data are deidentified, individual informed consent was not required. The research protocol received approval from the institutional review board at BIDMC and was implemented in accordance with the Declaration of Helsinki.

### 2.2. Study Population

**Figure 1:**
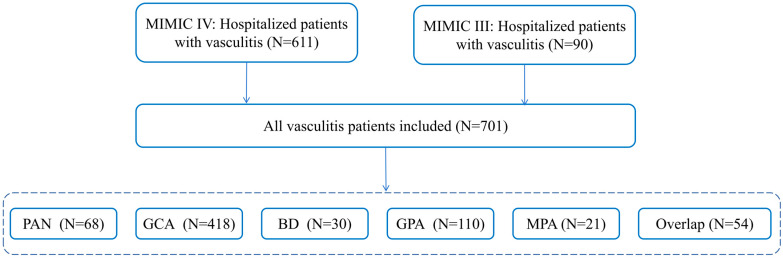
Flowchart of patient inclusion and exclusion from the MIMIC-III and MIMIC-IV databases. Abbreviations: GCA: giant cell arteritis; GPA: granulomatosis with polyangiitis; MPA: microscopic polyangiitis; PAN: polyarteritis nodosa; BD: Behçet’s disease; MIMIC-III: Medical Information Mart for Intensive Care-III; MIMIC-IV: Medical Information Mart for Intensive Care-IV.

Due to incomplete clinical data for outpatient cases of vasculitis, the present study included only hospitalized patients. Using the International Classification of Diseases (ICD) 9 and 10 codes, we retrospectively identified patients diagnosed with GCA (446.5 and M31.6), GPA (446.4, M31.30, and M31.31), PAN (446.0, M30.0, M30.1, and M30.8), BD (136.1), and MPA (M31.7) from the MIMIC-III and MIMIC-IV databases. BD was identified using ICD-9 code 136.1. No corresponding ICD-10 codes were present in the extracted dataset. For patients with multiple hospital admissions, only data from the first admission were included in the analysis. Patients under 18 years of age, those with incomplete medical records, or those diagnosed with other autoimmune diseases were excluded. The final study cohort comprised 611 patients from MIMIC-IV and 90 patients from MIMIC-III with confirmed vasculitis diagnoses (Table 1). Figure 1 illustrates the patient selection process and details the number of cases included for each disease. Overlap was defined as the presence of ICD codes corresponding to more than one vasculitis subtype during the same hospitalization.

### 2.3. Data Extraction and Definitions

Data were extracted from the MIMIC-IV and MIMIC-III databases using PostgreSQL software (version 13.4; PostgreSQL Global Development Group, Berkeley, CA, USA), with a focus on demographic characteristics, medication use, prognostic outcomes, comorbidities, and laboratory measurements. Demographic variables included age and sex, while laboratory parameters included tests related to complete blood count, liver function, renal function, and electrolyte levels. All laboratory variables, including BUN, were extracted from measurements obtained within the first 24 h of hospital admission to ensure temporal precedence relative to AKI development during hospitalization. The comorbidities considered in this study included AKI, hypertension, stroke, heart failure, and myocardial infarction. AKI was defined according to the KDIGO serum Cr criteria because urine output data were frequently incomplete in the MIMIC databases. The absence of urine output information may have resulted in underestimation of non-oliguric or transient AKI cases, potentially introducing misclassification bias. This limitation could affect both AKI incidence estimates and the observed associations between BUN, AKI, and mortality. Because BUN distributions differed between the overall vasculitis cohort and the subgroup of patients who developed AKI, tertile cut-offs were determined separately within each analytical population. Medication use was categorized into immunosuppressants, glucocorticoids (GCs), monoclonal antibody agents, and diuretics. The ICD codes used to identify each comorbidity are detailed in Supplementary Table S1, and the generic sequence numbers used for medication extraction are listed in Supplementary Table S2. To address missing data, continuous variables with a missing rate exceeding 15% were excluded from the analysis; however, no included variables met this criterion. Variables with a missing rate between 5% and 15% were imputed using multiple techniques. A summary of the variables with missing data included in this study is provided in Supplementary Table S3. The primary outcomes were defined as all-cause mortality at both short-term (30 days) and long-term (365 days) follow-up.

### 2.4. Statistical Analysis

Categorical variables were summarized as proportions. For continuous variables, distributional characteristics were first assessed. Variables exhibiting approximately normal distributions were presented as means with standard deviations, whereas those with skewed distributions were expressed as medians with interquartile ranges. Depending on the type and distribution of the data, appropriate statistical tests were employed for group comparisons, including Student’s *t*-test, the Mann–Whitney U test, the Chi-square test, and Fisher’s exact test. To identify the most relevant features associated with vasculitis, the least absolute shrinkage and selection operator (LASSO) regression was applied for variable selection. The variables selected by LASSO were subsequently incorporated into multivariable logistic regression or Cox proportional hazards models. Survival outcomes were evaluated using Kaplan–Meier (KM) survival curves along with log-rank tests. Restricted cubic splines (RCSs) were utilized to investigate potential nonlinear associations. All statistical analyses were conducted using R software (version 4.4.1; R Foundation for Statistical Computing, Vienna, Austria) and DecisionLinnc version 1.0 (Zhejiang, China) [31]. A two-sided *p*-value < 0.05 was considered statistically significant. The results of the logistic and Cox regression analyses were reported as odds ratios (ORs) or hazard ratios (HRs) with corresponding 95% confidence intervals (CIs).

### 2.5. Machine Learning

The dataset of vasculitis patients who developed AKI was randomly divided into a training set and a testing set at a 7:3 ratio. All data preprocessing steps were performed within the training set to avoid data leakage. Prior to model development, continuous variables were inspected for outliers and missingness. Missing values among candidate predictors were imputed using multivariate imputation with chained equations. Continuous variables were standardized using z-score normalization, and categorical variables were transformed via one-hot encoding. To address class imbalance in 365-day mortality outcomes, synthetic minority oversampling was applied exclusively within the training set, while the testing set remained untouched. Feature selection was conducted exclusively within the training set using a combined approach of LASSO regression and multivariable logistic regression. Predictors identified through this process, together with variable importance rankings, were used as input features for multiple machine learning algorithms, including decision tree (DT), random forest (RF), extreme gradient boosting (XGBoost), support vector machine (SVM), logistic regression (as a linear baseline), LightGBM, and k-nearest neighbors (KNN). Optimal hyperparameters were determined during model development using the training set. The testing set was reserved solely for independent performance evaluation and was not involved in any stage of feature selection, preprocessing parameter estimation, or hyperparameter tuning. Model performance was evaluated using receiver operating characteristic (ROC) curves and the corresponding area under the curve (AUC) values. To assess clinical utility, decision curve analysis (DCA) was also conducted. Model calibration was evaluated using calibration plots with LOESS smoothing and quantified using the Brier score. To facilitate clinical interpretation, threshold-dependent classification performance was evaluated on the testing set. The optimal probability cutoff was determined using the Youden index from the ROC curve, and the corresponding confusion matrix, sensitivity, specificity, and related metrics were reported. To enhance model interpretability, Shapley Additive Explanations (SHAP) were calculated for the final logistic regression model. Internal validation was performed using repeated bootstrap-like resampling with 100 iterations, each randomly sampling 70% of the dataset for model training. Model performance was summarized across resamples using ROC-based metrics, and 95% confidence intervals were derived to assess stability. The development and reporting of the prediction model adhered to the Transparent Reporting of a multivariable prediction model for Individual Prognosis Or Diagnosis (TRIPOD) statement.

## 3. Results

### 3.1. Baseline Characteristics

A total of 701 vasculitis patients met the inclusion criteria for this study, comprising 611 cases from the MIMIC-IV database and 90 cases from MIMIC-III (Figure 1). Baseline characteristics stratified by AKI occurrence and vasculitis subtype are presented in Table 1. Among the included patients, 176 developed AKI, while 525 did not. GCA accounted for the largest proportion of cases in this cohort. Patients with AKI exhibited a higher proportion of males, longer hospital stays, and increased short-, medium-, and long-term mortality rates compared to those without AKI. Furthermore, these patients were more likely to experience heart failure and showed elevated usage rates of immunosuppressants, monoclonal antibody therapies, and diuretics. The relatively low rate of recorded immunosuppressant use may reflect limitations in inpatient medication documentation within the MIMIC databases and the inability to capture outpatient or pre-admission therapy. Laboratory tests also revealed significantly higher levels of white blood cells, anion gap, potassium, Cr, and BUN in the AKI group (all *p* < 0.05). Conversely, patients without AKI had a higher prevalence of hypertension and demonstrated greater levels of hemoglobin, platelet count, total calcium, and sodium (all *p* < 0.05). The higher prevalence of hypertension in the non-AKI group may reflect differences in vasculitis subtype distribution and baseline clinical characteristics, particularly the predominance of elderly patients with GCA, in whom hypertension is common but renal involvement is less frequent compared with small-vessel vasculitides.

Table 2 provides baseline comparisons of patients with AKI stratified by 30- and 365-day survival outcomes. For the 30-day endpoint, 156 patients survived and 20 did not; for the 365-day endpoint, 128 survived while 48 patients died. Although the number of long-term events was smaller than that of short-term events, it remained sufficient for multivariable modeling according to the events-per-variable principle. In both short-term (30-day) and long-term (365-day) mortality analyses, non-survivors were significantly older and had higher diuretic use, elevated WBC counts, greater anion gap, and increased levels of Cr and BUN (all *p* < 0.05), while their hemoglobin and platelet counts were lower compared to those of survivors (*p* < 0.05). Additionally, for the 365-day mortality group, non-survivors had longer hospitalizations and were more likely to have experienced heart failure or stroke (*p* < 0.05).

### 3.2. Relationship Between Vasculitis and BUN Levels and Their Association with AKI Incidence

To identify risk factors associated with the development of AKI in patients with vasculitis, we performed multivariable and LASSO logistic regression analyses (Table 3, Supplementary Figure S1A,B). Both analyses consistently identified BUN as a significant risk factor for AKI in vasculitis patients (*p* < 0.05). In addition, we further examined the association between BUN levels and the incidence of AKI among vasculitis patients using logistic regression analysis. As shown in Table 4, BUN levels were significantly associated with AKI occurrence in vasculitis patients. Three models were constructed: Model 1 was unadjusted, and the covariate adjustments for Models 2 and 3 are detailed in Table 4. The analysis revealed a persistent positive association between BUN levels and AKI in vasculitis patients, with Model 3 yielding an OR of 1.03 (95% CI: 1.02–1.05, *p* < 0.0001). Subsequently, BUN levels were categorized into tertiles based on their distribution in this cohort (Q1: 12 mg/dL [IQR 10–14]; Q2: 21 mg/dL [IQR 19–23]; Q3: 44.5 mg/dL [IQR 32–67.5]), with the lowest tertile (Q1) serving as the reference group. Tertile cut-offs were based on the BUN distribution of the overall vasculitis cohort. The analysis demonstrated that the odds of developing AKI were significantly higher in the highest tertile (Q3) compared to Q1 (OR: 10.24, 95% CI: 6.17–17.78, *p* < 0.0001). After full adjustment in Model 3, patients in the highest BUN tertile remained at significantly increased risk of AKI compared to those in the lowest tertile (OR: 5.67, 95% CI: 3.00–11.04, *p* < 0.0001).

### 3.3. Association of BUN Levels and Risk Factors with Short- and Long-Term Mortality in Vasculitis Patients with AKI

To identify risk factors associated with short- and long-term mortality in vasculitis patients with AKI, we performed both multivariable and LASSO Cox regression analyses (Table 5, Supplementary Figure S2A–D). These analyses consistently indicated that BUN was an independent risk factor for both short- and long-term mortality in this patient population (all *p* < 0.05). In addition, logistic regression analysis was used to further examine the association between BUN levels and short- and long-term mortality in vasculitis patients with AKI. As shown in Table 6, BUN levels were significantly correlated with both 30- and 365-day mortality. Three models were constructed: Model 1 was unadjusted, and the covariate adjustments for Models 2 and 3 are detailed in Table 6. The analysis showed that in Model 3, BUN remained positively associated with both short- and long-term mortality (all *p* < 0.05). Furthermore, BUN levels were categorized into tertiles according to their distribution among vasculitis patients who developed AKI (Q1: 20 mg/dL [IQR 16–23]; Q2: 35 mg/dL [IQR 29.5–41]; Q3: 77 mg/dL [IQR 60.25–102]), with Q1 serving as the reference group. Tertile cut-offs were calculated according to the BUN distribution among patients with AKI. The analysis revealed that patients in the highest tertile (Q3) had markedly increased odds of 30- and 365-day mortality compared to those in Q1. Even after full adjustment in Model 3, patients in the highest BUN tertile remained at significantly higher risk of both short- and long-term mortality.

### 3.4. Nonlinear Analyses

**Figure 2:**
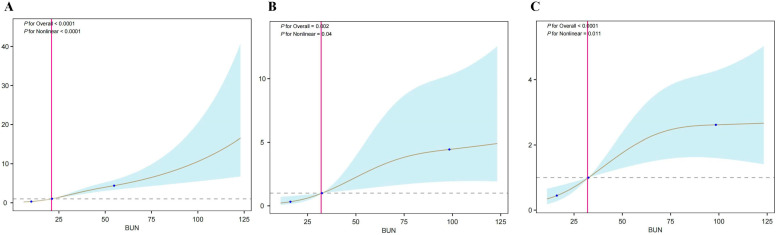
RCS analysis. (**A**) RCS analysis of BUN levels in relation to the occurrence of AKI in vasculitis patients; (**B**) RCS analysis of BUN levels and 30-day mortality in vasculitis patients with AKI. (**C**) RCS analysis of BUN levels and 365-day mortality in vasculitis patients with AKI. Abbreviations: BUN: blood urea nitrogen.

RCS analysis based on logistic regression revealed a significant nonlinear association between BUN levels and the occurrence of AKI in vasculitis patients (Figure 2A). Similarly, RCS analysis using Cox regression demonstrated significant nonlinear relationships between BUN levels and both 30- and 365-day mortality in vasculitis patients with AKI (Figure 2B,C). Threshold effect analysis of the RCS curves for short- and long-term mortality identified a critical BUN cutoff value of 32 mg/dL. This threshold reflects the inflection point of risk increase identified by spline modeling and should be interpreted as a statistical turning point rather than a definitive clinical decision threshold. Specifically, mortality risk remained relatively low when BUN levels were below this threshold; however, once BUN levels exceeded 32 mg/dL, the risk of death markedly increased.

### 3.5. KM Survival Analyses

**Figure 3:**
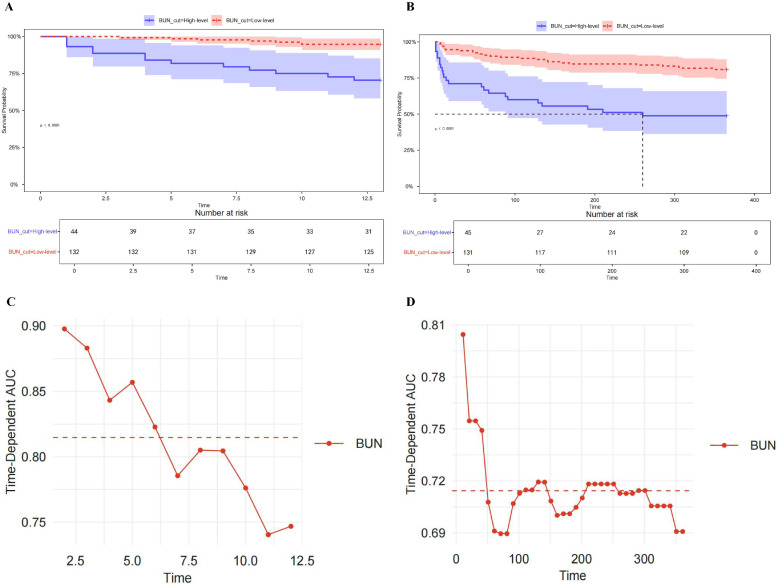
KM survival curves and time-dependent AUC curves. (**A**) KM survival curve of BUN levels for 30-day mortality in vasculitis patients with AKI; (**B**) KM survival curve of BUN levels for 365-day mortality in vasculitis patients with AKI; (**C**) time-dependent AUC curve of BUN levels for predicting 30-day mortality in vasculitis patients with AKI; (**D**) time-dependent AUC curve of BUN levels for predicting 365-day mortality in vasculitis patients with AKI. Abbreviations: AUC: area under the curve; BUN: blood urea nitrogen.

KM survival analysis was performed to evaluate 30- and 365-day mortality in vasculitis patients with AKI based on BUN levels. For 30-day mortality, the optimal BUN cutoff value was identified as 60 mg/dL, with 44 patients classified into the high BUN group and 132 into the low BUN group. For 365-day mortality, the optimal cutoff was 56 mg/dL, with 45 patients in the high BUN group and 131 in the low BUN group. These cut-off values were derived from data-driven optimization procedures and are intended for survival discrimination within this cohort rather than for universal clinical application. Patients with elevated BUN levels had significantly lower survival rates compared to those with lower BUN levels (*p* < 0.0001) (Figure 3A,B).

### 3.6. Time-Dependent AUC Curve

Figure 3C,D illustrate the time-dependent AUC curves for BUN in predicting 30- and 365-day mortality among vasculitis patients with AKI. For 30-day mortality, the AUC values remained consistently high throughout the time window (range: 0.85–0.90), indicating that BUN possesses strong and stable short-term prognostic performance. In contrast, for 365-day mortality, the AUC values were relatively lower (range: 0.69–0.81) and showed a slight decline over time, suggesting that the long-term predictive ability of BUN is comparatively weaker, though still within an acceptable range. These findings suggest that BUN is a more effective predictor of short-term outcomes in vasculitis patients with AKI.

### 3.7. The Results of Machine Learning

**Figure 4:**
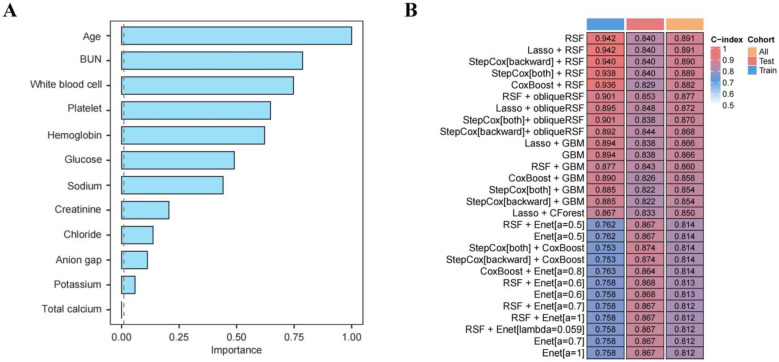
Comprehensive evaluation results of machine learning prognostic models. (**A**) Bar plot of variable importance rankings in the prognostic models; (**B**) heatmap of the comprehensive evaluation of prognostic models. Abbreviations: BUN: blood urea nitrogen.

**Figure 5:**
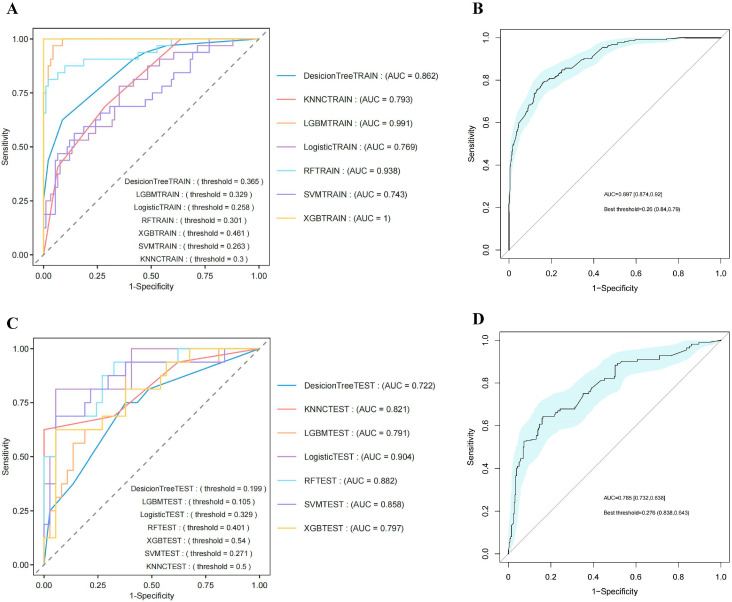
ROC curves for machine learning. (**A**) ROC curves of the training sets; (**B**) ROC curve of training sets with 95% CI; (**C**) ROC curves of testing sets; (**D**) ROC curve of testing sets with 95% CI. Abbreviations: ROC: receiver operating characteristic; AUC: area under the curve; RF: random forest; XGB: extreme gradient boosting survival learner; SVM: support vector machine; KNN: k-nearest neighbors; LGBM: LightGBM; CI: confidence interval.

**Figure 6:**
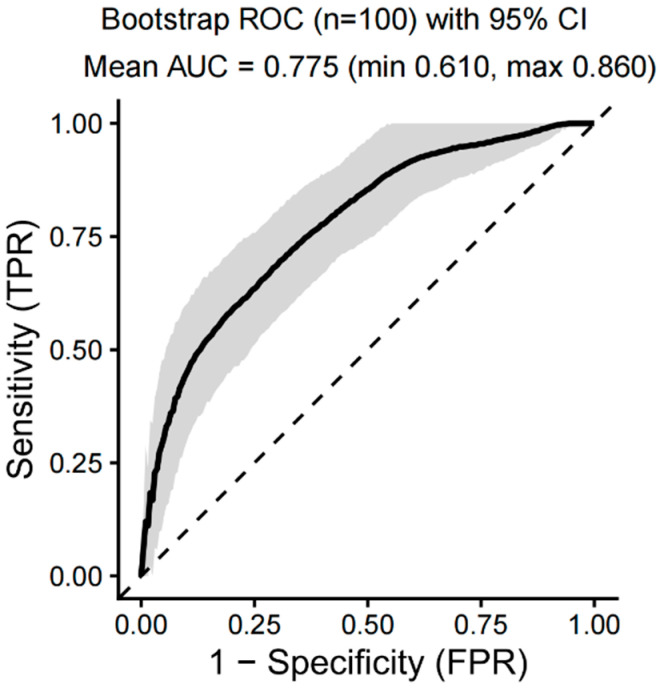
ROC curves of bootstrap analysis with 95% CI. Abbreviations: AUC: area under the curve; CI: confidence interval.

Machine learning was applied to predict 365-day mortality in vasculitis patients with AKI. When evaluating the prognostic models, the variable importance bar plot identified age and BUN as the most influential features, while the concordance index ranking in the evaluation heatmap indicated that the RF model had the highest predictive accuracy (Figure 4A,B). The optimal hyperparameters for all seven models are summarized in Supplementary Table S4. Figure 5 presents the ROC curves for both the training and testing sets. Although ensemble models such as XGBoost and LightGBM achieved high AUC values in the training set, their performance declined noticeably in the testing set, suggesting potential overfitting. In contrast, the logistic regression model demonstrated more stable performance across datasets and achieved the highest AUC in the testing set (0.904), indicating superior generalizability. Given its robustness, consistency between training and testing performance, and clinical interpretability, logistic regression was selected as the final model. DCA for the training and testing sets is shown in Supplementary Figure S3A and S3B, respectively, demonstrating consistent net clinical benefit. At clinically relevant threshold probabilities around 0.2–0.3, the logistic regression model achieved a net benefit of approximately 0.15–0.20 compared with treat-all and treat-none strategies. Calibration curves demonstrated good agreement between predicted and observed probabilities in both training and testing sets (Supplementary Figure S3C,D). On the independent testing set, the optimal probability threshold determined by the Youden index was 0.329. At this cutoff, the logistic regression model achieved a sensitivity of 0.812 and a specificity of 0.946, with an overall accuracy of 0.906, indicating strong discriminative performance. The detailed confusion matrix is shown in Supplementary Figure S4A,B. SHAP analysis further supported the relative importance of the selected predictors (Supplementary Figure S4C). Internal validation was performed using bootstrap resampling with 100 iterations. The bootstrap ROC curve with its corresponding 95% CIs is presented in Figure 6. Across 100 bootstrap resamples, the model demonstrated stable discrimination with a mean AUC of 0.775 (range: 0.610–0.860). The slightly lower bootstrap-estimated performance compared with the single testing-set AUC (0.904) reflects optimism correction and provides a more conservative estimate of internal validity. Bootstrap-based DCA and calibration analyses are shown in Supplementary Figure S3E,F, further supporting the stability and clinical applicability of the model. These findings highlight the importance of model parsimony and robustness, particularly in studies with relatively limited sample sizes.

## 4. Discussion

This study is the first to utilize a large public database to investigate the relationship between multiple types of vasculitis, AKI, and associated mortality, providing novel insights based on an analysis of 701 vasculitis patients. We found that AKI occurred in approximately 25% of vasculitis patients and was associated with significantly higher short-, medium-, and long-term mortality. Importantly, BUN emerged as a robust and independent predictor of both AKI occurrence and mortality in this population. Higher BUN levels were significantly associated with increased odds of AKI, even after adjusting for potential confounders, with patients in the highest tertile demonstrating a 5.67-fold higher risk compared to those in the lowest tertile. Furthermore, BUN was independently associated with both 30- and 365-day mortality in vasculitis patients with AKI, with mortality risk markedly increasing when BUN exceeded 32 mg/dL. Time-dependent AUC analysis demonstrated that BUN possesses strong predictive value for short-term mortality and acceptable performance for long-term outcomes. Additionally, machine learning models further underscored the prognostic significance of BUN and age, with the logistic regression model achieving the highest predictive accuracy. These findings highlight the clinical utility of BUN as a simple yet powerful biomarker for early risk stratification and prognosis in vasculitis patients, emphasizing the need for routine monitoring and timely interventions to mitigate adverse renal and mortality outcomes.

Renal involvement is a clinically significant complication across various types of systemic vasculitis, although its prevalence and manifestations differ by subtype. Pauci-immune necrotizing and crescentic glomerulonephritis is the hallmark renal lesion in small-vessel vasculitis such as MPA and GPA, occurring in 80–100% and 60–70% of cases, respectively. This pathology often leads to RPGN, AKI, and progression to end-stage renal disease (ESRD) if left untreated [5,32,33]. While MPA predominantly affects small vessels, GPA may also demonstrate granulomatous inflammation involving multiple organs [34]. Medium-vessel vasculitis, such as PAN, typically spares the glomeruli but affects medium-sized renal arteries, leading to ischemia, infarction, renovascular hypertension, and complications such as aneurysm formation and hemorrhage [7,22,35]. Although BD rarely involves the kidneys, reported lesions include glomerulonephritis, AA-type amyloidosis, and vascular lesions such as renal artery aneurysms and thrombosis. Most BD-associated renal involvement is mild, with microscopic hematuria or proteinuria, but severe complications like nephrotic syndrome and ESRD have been described [10,11,23]. In GCA, renal involvement is uncommon and typically results from renal artery or arteriolar inflammation, which can lead to ischemia, renal dysfunction, and rarely, fatal renal failure [8,36]. These observations highlight the spectrum renal pathology across vasculitis, emphasizing the importance of routinely monitoring renal function to enable early detection and intervention [36,37]. While previous studies on vasculitis have largely focused on individual subtypes or small cohorts and often lacked comprehensive renal outcome data, our study analyzed a large, heterogeneous population, providing novel epidemiological evidence on AKI incidence and its significant impact on mortality in vasculitis patients.

Previous studies have highlighted the clinical relevance of elevated BUN levels in vasculitis patients with renal involvement. In BD, the routine monitoring of BUN, alongside serum Cr, has been recommended for the early detection of renal impairment, as amyloidosis and glomerulonephritis are major contributors to renal failure in this population [23]. Similarly, in GPA, patients with RPGN demonstrated markedly increased BUN levels correlating with severe renal dysfunction and rapid progression to ESRD and death [24]. In PAN, BUN elevation was observed in the context of AKI, where vascular injury and hypoperfusion contributed to progressive renal deterioration, necessitating dialysis in refractory cases [22]. Although serum Cr and the estimated glomerular filtration rate (eGFR) remain the most commonly used biomarkers of renal function, they have important limitations in the acute setting. Cr elevation is often delayed in AKI, is strongly influenced by age, muscle mass, and chronic comorbidities, and may therefore underestimate early renal injury. These limitations are particularly evident in complex systemic conditions, such as cirrhosis, where altered muscle mass, fluid shifts, and inflammatory states further compromise Cr-based assessment of renal function [38]. Emerging evidence suggests that a multimarker approach incorporating both filtration and tubular injury biomarkers may improve diagnostic accuracy and prognostic stratification in AKI. Among filtration markers, cystatin C has shown greater independence from muscle mass and may provide improved GFR estimation in selected populations; however, it is not routinely measured in many real-world settings, including the MIMIC databases. Similarly, injury markers such as NGAL and KIM-1 have demonstrated potential for differentiating etiologies of AKI, but their availability and long-term validation remain limited in large heterogeneous cohorts. In contrast, BUN responds more rapidly to renal hypoperfusion, catabolic stress, and systemic inflammation—pathophysiologic mechanisms frequently observed in vasculitis—and its measurement is universally accessible in clinical practice. These characteristics make BUN a pragmatic and physiologically relevant marker for detecting early renal stress and predicting outcomes in heterogeneous, real-world vasculitis populations [39,40]. However, there are no reports specifically investigating BUN as a predictive marker for AKI in vasculitis. Our study builds upon these findings by analyzing a large, heterogeneous cohort of 701 vasculitis patients across multiple subtypes, providing a comprehensive assessment of BUN as a predictor of AKI and mortality. Importantly, we demonstrated that higher BUN levels were independently associated with increased odds of AKI as well as elevated short- and long-term mortality risks. The integration of advanced machine learning algorithms further reinforced the prognostic value of BUN and age, with logistic regression showing the most stable performance across datasets. These findings suggest that BUN may serve as a readily available and clinically accessible marker for risk assessment in vasculitis patients. Although multiple BUN thresholds (e.g., 32, 56, and 60 mg/dL) were identified using different analytical approaches, these values reflect method-specific statistical optimization rather than distinct biological cut-offs. Notably, the consistent pattern across models was a monotonic increase in risk with rising BUN levels. Therefore, BUN should primarily be interpreted as a continuous risk indicator, and clinical risk stratification may be more appropriately guided by model-based probability estimation rather than fixed categorical thresholds.

Recent advances in kidney disease prediction have increasingly leveraged deep learning architectures applied to imaging data. For example, the ConvNext-PCA framework integrates principal component analysis within convolutional neural networks to achieve highly efficient and accurate classification of kidney abnormalities from CT images [41]. While such approaches demonstrate impressive performance in image-based diagnosis, our study focused on structured clinical and laboratory data in critically ill vasculitis patients. In this context, simpler and more interpretable models such as logistic regression may offer advantages in terms of transparency, deployment feasibility, and compatibility with routine clinical workflows. Future research integrating multimodal data, including imaging and laboratory markers, may further enhance predictive accuracy.

This study has several limitations. First, its retrospective design and reliance on the MIMIC-III and MIMIC-IV databases may introduce selection bias and limit the generalizability, particularly given that the cohort was predominantly composed of patients with GCA. As different vasculitis subtypes may involve distinct mechanisms of renal injury, caution is warranted when extrapolating these findings across all vasculitis categories. Second, vasculitis cases and comorbidities were identified using ICD-9/10 codes, which may introduce coding bias, and the databases lack detailed information on disease activity, immunosuppressive regimens, renal pathology, baseline CKD stage, and fluid management. Third, AKI was defined solely according to the KDIGO serum Cr criteria because urine output data were frequently incomplete, potentially leading to underestimation of non-oliguric AKI and misclassification of mild or transient cases, which may have biased effect estimates toward the null. Baseline and longitudinal renal function data were unavailable, limiting clinical characterization. In addition, the higher prevalence of hypertension in the non-AKI group may reflect differences in vasculitis subtype distribution and age-related comorbidity. Moreover, the observational design precludes definitive causal inference regarding the relationships among BUN, AKI, and mortality. Although baseline BUN values were extracted at admission, the exact temporal relationship between BUN elevation and subsequent AKI onset cannot be fully determined, and reverse causation cannot be completely excluded. Additionally, BUN levels may be influenced by fluid status and catabolic conditions, which were not fully captured in the database and may introduce residual confounding. In addition, the relatively limited number of long-term mortality events may reduce statistical power and affect the stability of estimates for 365-day outcomes. Medication exposure was based on inpatient records and may underestimate prior or outpatient immunosuppressive therapy. Finally, despite multivariable adjustment, residual confounding from unmeasured variables remains possible. External validation is required to confirm model robustness.

## 5. Conclusions

This study demonstrates that BUN is an independent predictor of AKI and mortality in vasculitis patients. Using a large, heterogeneous cohort and machine learning approaches, we provide robust evidence supporting BUN as a practical biomarker for early risk stratification and improved clinical management. Given that the BUN thresholds identified in this study were derived from ICU-based data, future prospective studies and external validation in non-ICU vasculitis cohorts are required to confirm their generalizability and clinical applicability.

## Figures and Tables

**Table 1:** The clinical characteristics of all vasculitis patients stratified by the presence or absence of AKI.

Variable	Overall (*n* = 701)	Without AKI (*n* = 525)	AKI (*n* = 176)	*p*-Value
Age, years	75.76 (65.00–83.00)	76.00 (66.00–83.00)	74.00 (64.00–83.00)	0.4
Gender, *n* (%)				**0.004**
Female	445.00 (63.48%)	349.00 (66.48%)	96.00 (54.55%)	
Male	256.00 (36.52%)	176.00 (33.52%)	80.00 (45.45%)	
LOS of hospital, day	4.66 (2.50–7.89)	3.88 (2.15–6.95)	7.03 (3.87–13.00)	**<0.001**
Hospital mortality, n (%)				**<0.001**
No	668.00 (95.29%)	512.00 (97.52%)	156.00 (88.64%)	
Yes	33.00 (4.71%)	13.00 (2.48%)	20.00 (11.36%)	
30-day mortality, n (%)				**<0.001**
No	660.00 (94.15%)	504.00 (96.00%)	156.00 (88.64%)	
Yes	41.00 (5.85%)	21.00 (4.00%)	20.00 (11.36%)	
60-day mortality, n (%)				**0.001**
No	639.00 (91.16%)	489.00 (93.14%)	150.00 (85.23%)	
Yes	62.00 (8.84%)	36.00 (6.86%)	26.00 (14.77%)	
90-day mortality, n (%)				**<0.001**
No	626.00 (89.30%)	481.00 (91.62%)	145.00 (82.39%)	
Yes	75.00 (10.70%)	44.00 (8.38%)	31.00 (17.61%)	
180-day mortality, n (%)				**<0.001**
No	602.00 (85.88%)	466.00 (88.76%)	136.00 (77.27%)	
Yes	99.00 (14.12%)	59.00 (11.24%)	40.00 (22.73%)	
365-day mortality, n (%)				**0.002**
No	565.00 (80.60%)	437.00 (83.24%)	128.00 (72.73%)	
Yes	136.00 (19.40%)	88.00 (16.76%)	48.00 (27.27%)	
Hypertension, n (%)				**0.015**
No	407.00 (58.06%)	291.00 (55.43%)	116.00 (65.91%)	
Yes	294.00 (41.94%)	234.00 (44.57%)	60.00 (34.09%)	
Heart failure, n (%)				**0.047**
No	540.00 (77.03%)	414.00 (78.86%)	126.00 (71.59%)	
Yes	161.00 (22.97%)	111.00 (21.14%)	50.00 (28.41%)	
Myocardial infarction, n (%)				0.46
No	686.00 (97.86%)	515.00 (98.10%)	171.00 (97.16%)	
Yes	15.00 (2.14%)	10.00 (1.90%)	5.00 (2.84%)	
Stroke, n (%)				0.22
No	637.00 (90.87%)	473.00 (90.10%)	164.00 (93.18%)	
Yes	64.00 (9.13%)	52.00 (9.90%)	12.00 (6.82%)	
Immunosuppressants, n (%)				**0.021**
No	626.00 (89.30%)	477.00 (90.86%)	149.00 (84.66%)	
Yes	75.00 (10.70%)	48.00 (9.14%)	27.00 (15.34%)	
GCs, n (%)				0.72
No	303.00 (43.22%)	229.00 (43.62%)	74.00 (42.05%)	
Yes	398.00 (56.78%)	296.00 (56.38%)	102.00 (57.95%)	
Monoclonal antibody agent, n (%)				**<0.001**
No	664.00 (94.72%)	509.00 (96.95%)	155.00 (88.07%)	
Yes	37.00 (5.28%)	16.00 (3.05%)	21.00 (11.93%)	
Diuretics, n (%)				**0.007**
No	430.00 (61.34%)	337.00 (64.19%)	93.00 (52.84%)	
Yes	271.00 (38.66%)	188.00 (35.81%)	83.00 (47.16%)	
White blood cell, K/µL	9.00 (6.60–12.10)	8.60 (6.50–11.60)	9.50 (6.75–12.30)	**0.032**
Hemoglobin, g/dL	10.70 (9.30–12.10)	10.90 (9.70–12.20)	10.05 (8.65–11.50)	**<0.001**
Platelet, K/µL	239.64 (182.00–311.00)	244.00 (185.00–321.00)	235.50 (168.50–287.00)	**0.023**
Anion gap, mmol/L	14.00 (12.00–16.00)	14.00 (12.00–16.00)	15.00 (13.00–18.00)	**<0.001**
Total calcium, mmol/L	8.70 (8.30–9.20)	8.80 (8.30–9.20)	8.60 (8.05–9.10)	**<0.001**
Chloride, mmol/L	103.00 (100.00–106.00)	103.00 (99.00–106.00)	103.00 (100.00–107.00)	0.092
Glucose, mg/dL	109.00 (90.00–148.00)	109.00 (90.00–147.00)	115.00 (91.00–153.50)	0.44
Potassium, mmol/L	4.10 (3.80–4.50)	4.00 (3.70–4.40)	4.30 (3.90–4.90)	**<0.001**
Sodium, mmol/L	139.00 (136.00–141.00)	139.00 (137.00–141.00)	138.00 (136.00–141.00)	**0.018**
Cr, mg/dL	1.00 (0.70–1.50)	0.90 (0.70–1.10)	1.65 (1.20–2.85)	**<0.001**
BUN, mg/dL	21.00 (14.00–32.00)	18.00 (13.00–26.00)	32.50 (22.50–58.00)	**<0.001**

AKI: acute kidney injury; GCs: glucocorticoids; Cr: Creatinine; BUN: Blood urea nitrogen. Significant *p* values are in bold.

**Table 2:** The clinical characteristics of vasculitis patients with AKI in relation to short- and long-term mortality.

Variable	Overall (*n* = 176)	30-Day Mortality			365-Day Mortality		
		Survivors (*n* = 156)	Non-Survivors (*n* = 20)	*p*-Value	Survivors (*n* = 128)	Non-Survivors (*n* = 48)	*p*-Value
Age, years	74.00 (64.00–83.00)	73.00 (63.00–81.01)	81.00 (72.99–86.31)	**0.011**	71.00 (60.56–79.32)	81.50 (71.67–87.58)	**<0.001**
Gender, *n* (%)				0.97			0.16
Female	96.00 (54.55%)	85.00 (54.49%)	11.00 (55.00%)		74.00 (57.81%)	22.00 (45.83%)	
Male	80.00 (45.45%)	71.00 (45.51%)	9.00 (45.00%)		54.00 (42.19%)	26.00 (54.17%)	
LOS of hospital, day	7.03 (3.87–13.00)	6.87 (3.90–13.28)	7.79 (3.54–12.54)	0.91	6.54 (3.42–12.24)	9.68 (4.47–15.68)	**0.038**
Hypertension, n (%)				0.93			0.4
No	116.00 (65.91%)	103.00 (66.03%)	13.00 (65.00%)		82.00 (64.06%)	34.00 (70.83%)	
Yes	60.00 (34.09%)	53.00 (33.97%)	7.00 (35.00%)		46.00 (35.94%)	14.00 (29.17%)	
Heart failure, n (%)				0.22			**0.017**
No	126.00 (71.59%)	114.00 (73.08%)	12.00 (60.00%)		98.00 (76.56%)	28.00 (58.33%)	
Yes	50.00 (28.41%)	42.00 (26.92%)	8.00 (40.00%)		30.00 (23.44%)	20.00 (41.67%)	
Myocardial infarction, n (%)				0.54			0.52
No	171.00 (97.16%)	152.00 (97.44%)	19.00 (95.00%)		125.00 (97.66%)	46.00 (95.83%)	
Yes	5.00 (2.84%)	4.00 (2.56%)	1.00 (5.00%)		3.00 (2.34%)	2.00 (4.17%)	
Stroke, n (%)				0.55			**0.012**
No	164.00 (93.18%)	146.00 (93.59%)	18.00 (90.00%)		123.00 (96.09%)	41.00 (85.42%)	
Yes	12.00 (6.82%)	10.00 (6.41%)	2.00 (10.00%)		5.00 (3.91%)	7.00 (14.58%)	
Immunosuppressants, n (%)				0.2			0.44
No	149.00 (84.66%)	134.00 (85.90%)	15.00 (75.00%)		110.00 (85.94%)	39.00 (81.25%)	
Yes	27.00 (15.34%)	22.00 (14.10%)	5.00 (25.00%)		18.00 (14.06%)	9.00 (18.75%)	
GCs, n (%)				0.78			0.15
No	74.00 (42.05%)	65.00 (41.67%)	9.00 (45.00%)		58.00 (45.31%)	16.00 (33.33%)	
Yes	102.00 (57.95%)	91.00 (58.33%)	11.00 (55.00%)		70.00 (54.69%)	32.00 (66.67%)	
Monoclonal antibody agent, n (%)				0.31			0.89
No	155.00 (88.07%)	136.00 (87.18%)	19.00 (95.00%)		113.00 (88.28%)	42.00 (87.50%)	
Yes	21.00 (11.93%)	20.00 (12.82%)	1.00 (5.00%)		15.00 (11.72%)	6.00 (12.50%)	
Diuretics, n (%)				**0.002**			**0.005**
No	93.00 (52.84%)	89.00 (57.05%)	4.00 (20.00%)		76.00 (59.38%)	17.00 (35.42%)	
Yes	83.00 (47.16%)	67.00 (42.95%)	16.00 (80.00%)		52.00 (40.63%)	31.00 (64.58%)	
White blood cell, K/µL	9.50 (6.75–12.30)	9.40 (6.70–12.05)	13.45 (9.95–26.90)	**0.003**	9.35 (6.55–11.75)	11.35 (7.95–16.10)	**0.006**
Hemoglobin, g/dL	10.05 (8.65–11.50)	10.15 (8.80–11.75)	8.75 (7.95–9.30)	**0.002**	10.25 (9.00–11.80)	9.00 (7.95–10.55)	**0.001**
Platelet, K/µL	235.50 (168.50–287.00)	236.00 (179.00–291.50)	171.50 (127.50–242.00)	**0.011**	236.00 (180.00–284.00)	221.00 (144.00–297.00)	0.19
Anion gap, mmol/L	15.00 (13.00–18.00)	15.00 (12.00–17.00)	17.00 (14.00–20.00)	**0.014**	14.00 (12.00–17.00)	16.00 (13.00–18.50)	**0.025**
Total calcium, mmol/L	8.60 (8.05–9.10)	8.60 (8.00–9.10)	8.35 (8.10–8.75)	0.4	8.60 (8.10–9.10)	8.40 (8.00–8.95)	0.38
Chloride, mmol/L	103.00 (100.00–107.00)	103.00 (100.00–107.00)	104.50 (99.00–107.50)	0.83	103.50 (101.00–107.00)	103.00 (98.50–106.00)	0.33
Glucose, mg/dL	115.00 (91.00–153.50)	115.50 (91.50–151.00)	108.50 (84.50–160.00)	0.88	116.00 (90.00–155.00)	106.50 (96.50–144.50)	0.68
Potassium, mmol/L	4.30 (3.90–4.90)	4.30 (3.90–4.90)	4.60 (4.10–5.00)	0.14	4.30 (3.80–4.90)	4.30 (4.00–4.85)	0.56
Sodium, mmol/L	138.00 (136.00–141.00)	138.00 (136.00–140.50)	140.50 (135.50–143.00)	0.13	138.00 (136.00–140.50)	138.50 (136.00–141.00)	0.39
Cr, mg/dL	1.65 (1.20–2.85)	1.60 (1.15–2.55)	2.85 (1.40–3.95)	**0.033**	1.55 (1.15–2.30)	2.45 (1.20–3.95)	**0.018**
BUN, mg/dL	32.50 (22.50–58.00)	31.50 (22.00–51.00)	65.00 (45.50–109.00)	**<0.001**	29.50 (21.00–48.00)	52.00 (28.00–87.00)	**<0.001**

AKI: acute kidney injury; GCs: glucocorticoids; Cr: Creatinine; BUN: Blood urea nitrogen. Significant *p* values are in bold.

**Table 3:** Logistic analysis: incidence of AKI among vasculitis patients.

Variables	Multivariate Logistic Analysis		LASSO-Logistic Analysis	
	ORs (95% CIs)	*p*-Value	ORs (95% CIs)	*p*-Value
Gender	1.54 (1.00–2.36)	**0.047**		
Monoclonal antibody agent	3.68 (1.62–8.44)	**0.** **002**	3.66 (1.62–8.30)	**0.002**
Chloride	1.10 (1.04–1.17)	**0.** **003**	1.10 (1.04–1.16)	**0.** **002**
Potassium	1.60 (1.16–2.24)	**0.** **005**	1.61 (1.16–2.24)	**0.** **004**
Sodium	0.91 (0.84–0.98)	**0.01**	0.92 (0.86–0.99)	**0.02**
BUN	1.03 (1.02–1.05)	**<0.0001**	1.03 (1.02–1.04)	**<0.0001**

AKI: acute kidney injury; LASSO: least absolute shrinkage and selection operator; ORs: odds ratio; CIs: confidence intervals; BUN: blood urea nitrogen. Significant *p* values are in bold.

**Table 4:** The association between BUN levels and the occurrence of AKI in vasculitis patients by logistic regression analyses.

		Model 1		Model 2		Model 3	
		ORs (95% CIs)	*p*-Value	ORs (95% CIs)	*p*-Value	ORs (95% CIs)	*p*-Value
BUN		1.04 (1.03–1.05)	**<0.0001**	1.04 (1.03–1.05)	**<0.0001**	1.03 (1.02–1.05)	**<0.0001**
BUN (tertile)							
Q1	12 (10–14)	reference		reference		reference	
Q2	21 (19–23)	2.57 (1.49–4.59)	**0.0009**	2.72 (1.55–4.92)	**0.0006**	2.08 (1.15–3.87)	**0.02**
Q3	44.5 (32–67.5)	10.24 (6.17–17.78)	**<0.0001**	10.37 (6.12–18.36)	**<0.0001**	5.67 (3.00–11.04)	**<0.0001**
*p* for trend		<**0.0001**		**<0.0001**		**<0.0001**	

ORs: odds ratio; CIs: confidence intervals; Model 1: no covariates were adjusted. Model 2: adjusted for age, gender, hypertension, heart failure, myocardial infarction, stroke. Model 3: adjusted for age, gender, hypertension, heart failure, myocardial infarction, stroke, immunosuppressants, GC, monoclonal antibody agent, diuretics, white blood cell, hemoglobin, platelet, anion gap, total calcium, chloride, glucose, potassium, sodium, Cr. BUN: blood urea nitrogen; AKI: acute kidney injury. Significant *p* values are in bold.

**Table 5:** Cox analysis: risk factors for short- and long-term mortality in vasculitis patients with AKI.

Variables	Multivariate Cox Analysis		LASSO-Cox Analysis	
	HRs (95% CIs)	*p*-Value	HRs (95% CIs)	*p*-Value
30-day mortality				
Age	1.12 (1.05–1.21)	**0.001**	1.10 (1.04–1.17)	**0.002**
Diuretics	5.17 (1.24–21.50)	**0.024**		
White blood cell	1.08 (1.02–1.14)	**0.009**	1.08 (1.04–1.13)	**<0.0001**
Hemoglobin			0.73 (0.56–0.97)	**0.** **03**
BUN	1.03 (1.01–1.05)	**0.005**	1.02 (1.01–1.04)	**<0.0001**
365-day mortality				
Age	1.08 (1.04–1.12)	**<0.0001**	1.08 (1.05–1.12)	**<0.0001**
Diuretics			2.01 (1.09–3.71)	**0.025**
White blood cell	1.06 (1.02–1.10)	**0.002**	1.05 (1.02–1.08)	**<0.0001**
Hemoglobin	0.78 (0.64–0.95)	**0.** **013**	0.79 (0.67–0.93)	**0.** **005**
BUN	1.02 (1.00–1.03)	**0.** **008**	1.02 (1.00–1.02)	**<0.0001**

AKI: acute kidney injury; LASSO: least absolute shrinkage and selection operator; HRs: hazard ratios; CIs: confidence intervals; BUN: blood urea nitrogen. Significant *p* values are in bold.

**Table 6:** The association between BUN levels and short- and long-term mortality in vasculitis patients with AKI by logistic regression analysis.

		Model 1		Model 2		Model 3	
		ORs (95% CIs)	*p*-Value	ORs (95% CIs)	*p*-Value	ORs (95% CIs)	*p*-Value
30-day mortality							
BUN		1.02 (1.01–1.03)	**0.001**	1.03 (1.01–1.04)	**0.0002**	1.03 (1.01–1.04)	**0.0002**
BUN (tertile)							
Q1	20 (16–23)	reference		reference		reference	
Q2	35 (29.5–41)	0.75 (0.10–4.72)	0.76	0.67 (0.08–4.27)	0.67	0.62 (0.08–4.04)	0.62
Q3	77 (60.25–102)	6.98 (2.14–31.47)	**0.0034**	8.14 (2.36–38.50)	**0.0024**	8.30 (2.33–40.56)	**0.0028**
*p* for trend		**0.001**		**0.0008**		**0.0009**	
365-day mortality							
BUN		1.02 (1.01–1.03)	**0.0011**	1.02 (1.01–1.03)	**0.0001**	1.02 (1.01–1.03)	0.0002
BUN (tertile)							
Q1	20 (16–23)	reference		reference		reference	
Q2	35 (29.5–41)	1.33 (0.51–3.46)	0.56	1.24 (0.45–3.47)	0.67	1.24 (0.44–3.55)	0.69
Q3	77 (60.25–102)	4.62 (2.02–11.22)	**0.0001**	6.34 (2.50–17.65)	**0.0002**	6.12 (2.37–17.34)	**0.0003**
*p* for trend		**0.0003**		**0.0001**		**0.0002**	

ORs: odds ratio; CIs: confidence intervals; Model 1: no covariates were adjusted; Model 2: adjusted for age, gender; Model 3: adjusted for age, gender, hypertension, heart failure, myocardial infarction, stroke. AKI: acute kidney injury; BUN: blood urea nitrogen. Significant *p* values are in bold.

## Data Availability

The data of the present study were obtained from MIMIC-III (version 1.4) and MIMIC-IV (version 3.1). The availability of these data is restricted, and a license is needed. However, data are available from the author Si Chen (si.chen.anzhen@gmail.com) upon reasonable request and with approval from the Medical Information Mart for Intensive Care Institute.

## Supplementary Materials

The following supporting information can be downloaded at: https://www.mdpi.com/article/10.3390/diagnostics16050665/s1, for Supplementary Figures S1–S4 and Supplementary Tables S1–S4; see the Supplementary Materials. Figure S1. Identification of factors associated with AKI in vasculitis patients using LASSO binary logistic regression. Figure S2. Application of LASSO survival regression to identify factors associated with mortality in vasculitis patients with AKI. Figure S3. DCA and calibration curves. Figure S4. Performance evaluation and interpretability of the logistic regression model in the testing set. Table S1. ICD codes for vasculitis complications. Table S2. GSNs for drugs used in patients with vasculitis. Table S3. Missing values for all included variables in patients with vasculitis. Table S4. Hyperparameter of the nine models.
