# Supplementary material for: Prognostic Value of Blood Urea Nitrogen for Acute Kidney Injury and Mortality in Vasculitis: A Large Cohort Study Using Multivariate Joint Model and Machine Learning
Source: Diagnostics (Basel). 2026 Feb 25;16(5):665. doi: 10.3390/diagnostics16050665 (PMC12985082; doi:10.3390/diagnostics16050665)
Supplement: Supplementary file 1 [file diagnostics-16-00665-s001.zip › Supplementary Table.pdf]

Supplementary Table 1. ICD codes for vasculitis complications

Diseases	ICD-9/10
Hypertension	4019, I10, 4011, I161, 4010
Stroke	V1254, Z8673, 431, 43820, 43811, 4359, V171, 43883, 99702, G459
Heart failure	4280, 42832, 42822, I5032, 42833, I5033, I5022, 42823, I5023, I509, 42830, 42843, 42831, I5030, 42821, 42842, I5021, I5020, 42820, I5031, I5043, I5042, 40491, 40291, 42841, 4289, 42840, I5084, I50810, I5041, I5082, I5040, 4281, I50814, I50811, I50813, I50812, 40201, 40492, I5089, I5083
Myocardial infarction	41000, 41001, 41002, 41010, 41011, 41012 ,41020, 41021, 41022, 41030, 41031, 41032, 41040, 41041, 41042, 41050, 41051, 41052, 41080, 41081, 41082, 41090, 41091, 41092, I21, I219, I230, I231, I232, I233, I234, I235, I236, I238, I210, I2101, I2102, I2109, I211, I2111, I2119, I2121, I2129, I213, I214, I21A1, I21A9, I222
AKI	5849, N179, 5845, N170

ICD: International Classification of Diseases; AKI: acute kidney injury.

Supplementary Table 2. GSNs for drugs used in patients with vasculitis.

Drugs	GSNs
GCs	066110, 006705, 051558, 006704, 007544, 023906, 006696, 006858, 007545, 007543, 006724, 006725, 006753, 007894, 007892, 006786, 006784, 006788, 006776, 006778, 006789, 006721, 067556, 047282, 006745, 060958, 062053, 006780, 013701, 006762, 006758, 006812, 066112, 026721, 006749, 006738, 006742, 006754, 006748, 006750
Immunosuppressants	008765, 016500, 008771, 071489, 021796, 021797, 041832, 047347, 032599, 023724, 041845, 040376, 011682, 011681, 040549, 040550
Monoclonal antibody agent	036870, 065411, 079334, 071056, 071590, 080059
Diuretics	021408, 008209, 008205, 029832, 008163, 006816, 051036, 008151, 021409, 008210, 008213, 006817, 008166, 028915, 008227, 021718, 008217, 008201, 008223, 008182, 021406, 008148, 051037, 008183, 008221, 008224, 021410, 066298, 008206, 021407, 006818, 008149

GSNs: generic sequence numbers; GCs: glucocorticoids.

Supplementary Table 3. Missing values for all included variables in patients with vasculitis.

Variables	Missing
Hemoglobin	7.38%
Platelet	7.81%
White blood cell	7.38%
Anion gap	7.11%
Total calcium	10.65%
Chloride	7.03%
Glucose	7.03%
Potassium	7.03%
Sodium	7.03%
BUN	7.03%
Creatinine	7.03%

BUN: Blood urea nitrogen.

Supplementary Table 4. Hyperparameter of the nine models.

Model	Hyperparameter	Optimal hyperparameter
DT	Random Seed	1
	Splitting Criterion	best
	Loss Function	gini impurity
	Maximum Depth	3
	Minimum Samples for Split	2
	Minimum Samples for Leaf Nodes	1
	Minimum Impurity Decrease Threshold	0
	Random Seed	1

RF	Random Seed	1
	Number of Trees	100
	Loss Function	gini impurity
	Maximum Depth	3
	Minimum Samples for Split	2
	Minimum Samples for Leaf Nodes	1
	Minimum Impurity Decrease Threshold	0
XGBoost	Random Seed	1
	Weak Learner Type	gradient boosted decision tree
SVM	Random Seed	1
	Regularization Parameter	1
	Kernel Function	radial basis function
LightGBM	Set random seed	1
	Weak learner type	gbdt
	Learning rate	0.1
Logistic Classification	Set random seed	1
	Regularization parameter	1
	Penalty type selection	none
	Optimization algorithm selection	lbfgs
	Set tolerance	0.0001
	Set maximum iterations	100
k-Nearest Neighbors	Weight	uniform
	Number of neighbors	5
	Neighbor algorithm	auto

DT: decision tree; RF: random forest; XGBoost: extreme gradient boosting survival learner; SVM: support vector machine.
