# Supplementary figures and images for: Prognostic Value of Blood Urea Nitrogen for Acute Kidney Injury and Mortality in Vasculitis: A Large Cohort Study Using Multivariate Joint Model and Machine Learning
Source: Diagnostics (Basel). 2026 Feb 25;16(5):665. doi: 10.3390/diagnostics16050665 (PMC12985082; doi:10.3390/diagnostics16050665)

A

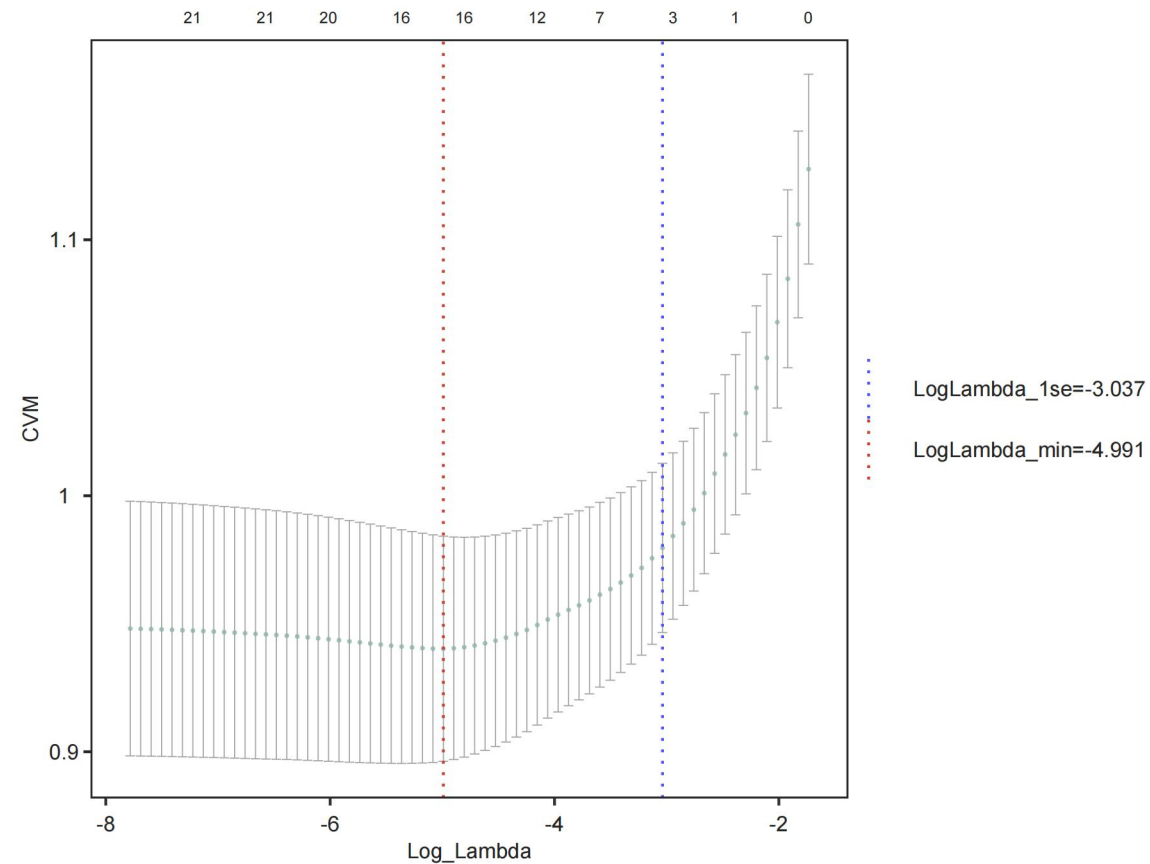

B

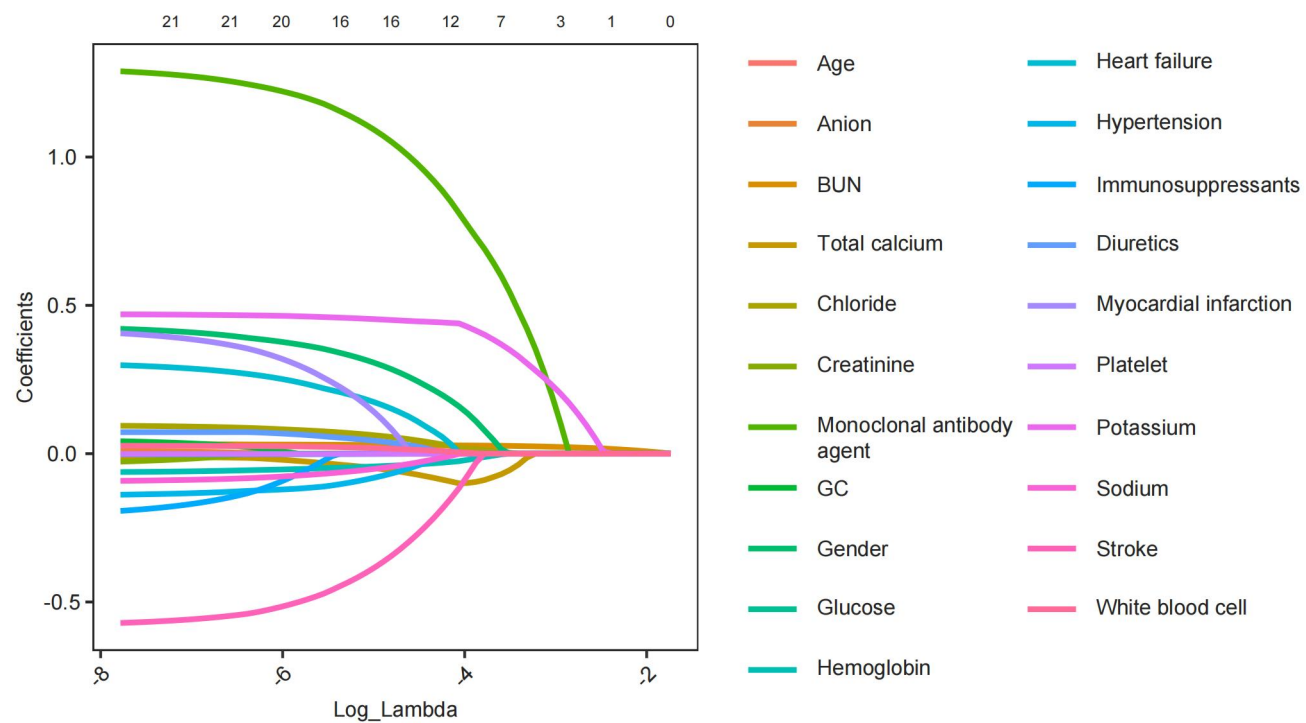

Supplement: Supplementary file 1 [file diagnostics-16-00665-s001.zip › Figure S1.pdf]

**A**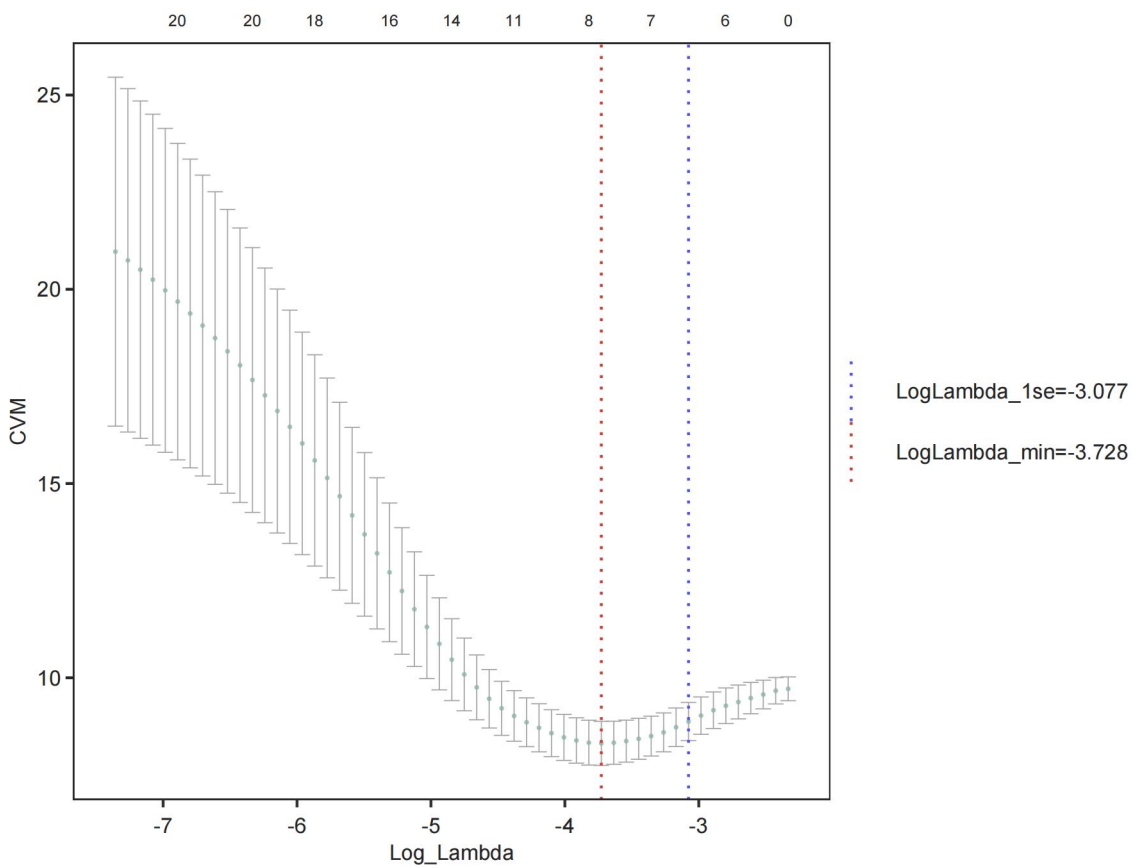**B**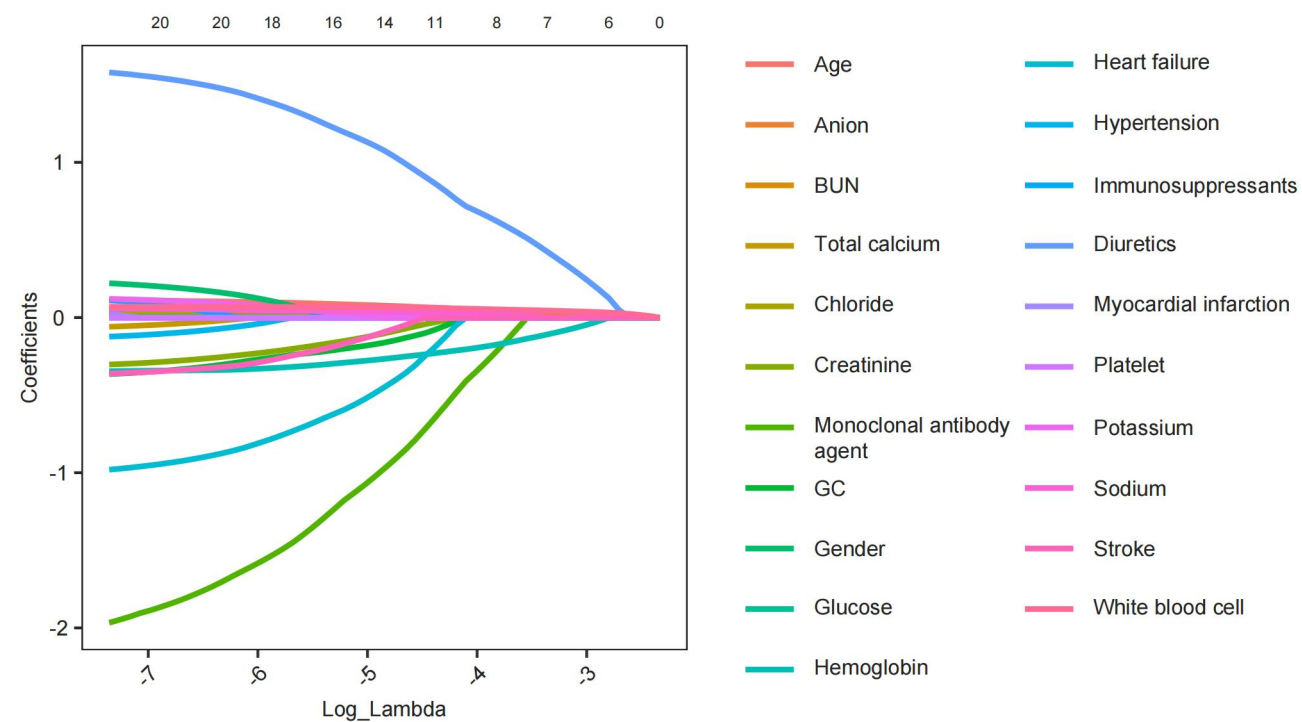**C**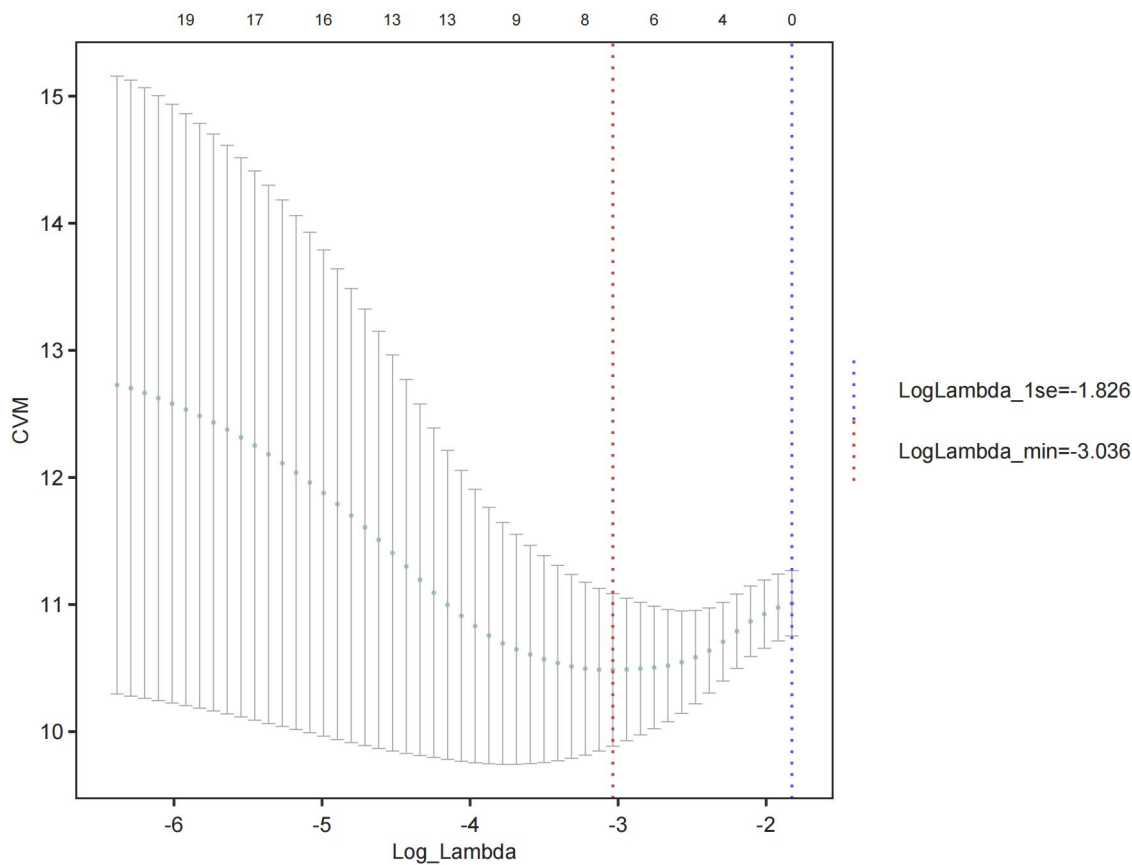**D**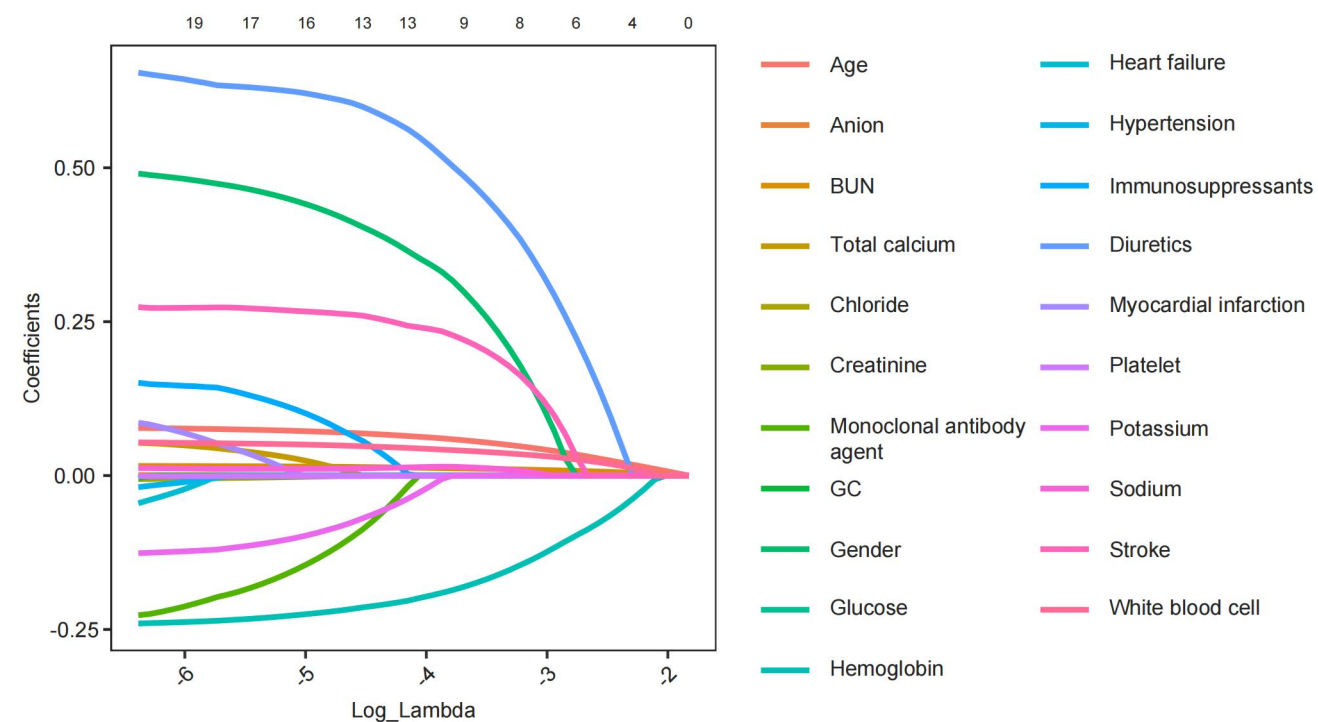

Supplement: Supplementary file 1 [file diagnostics-16-00665-s001.zip › Figure S2.pdf]

**A**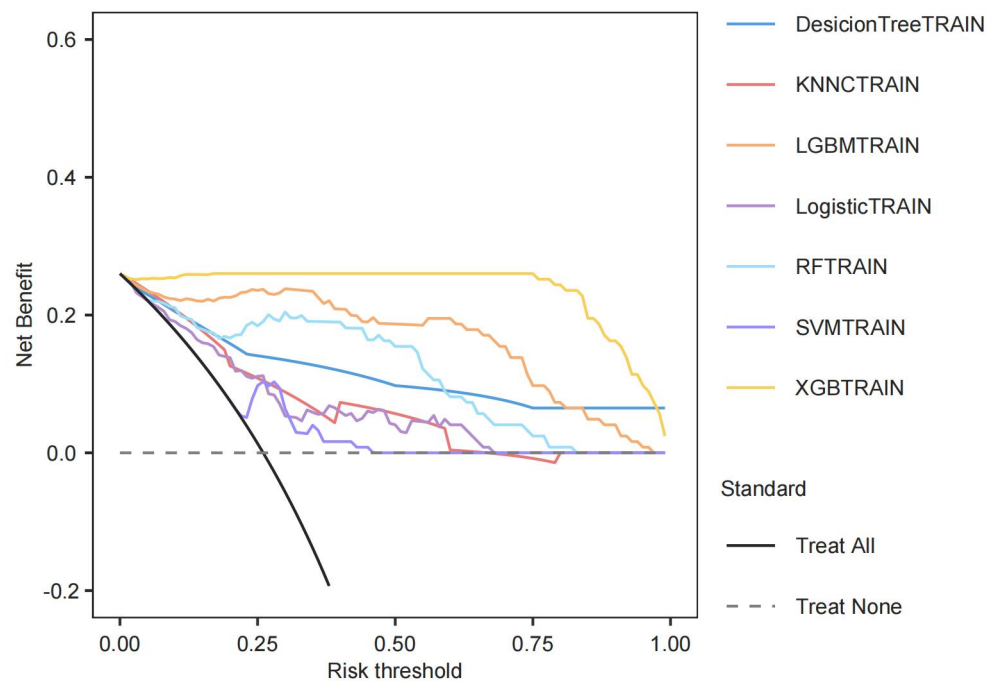**B**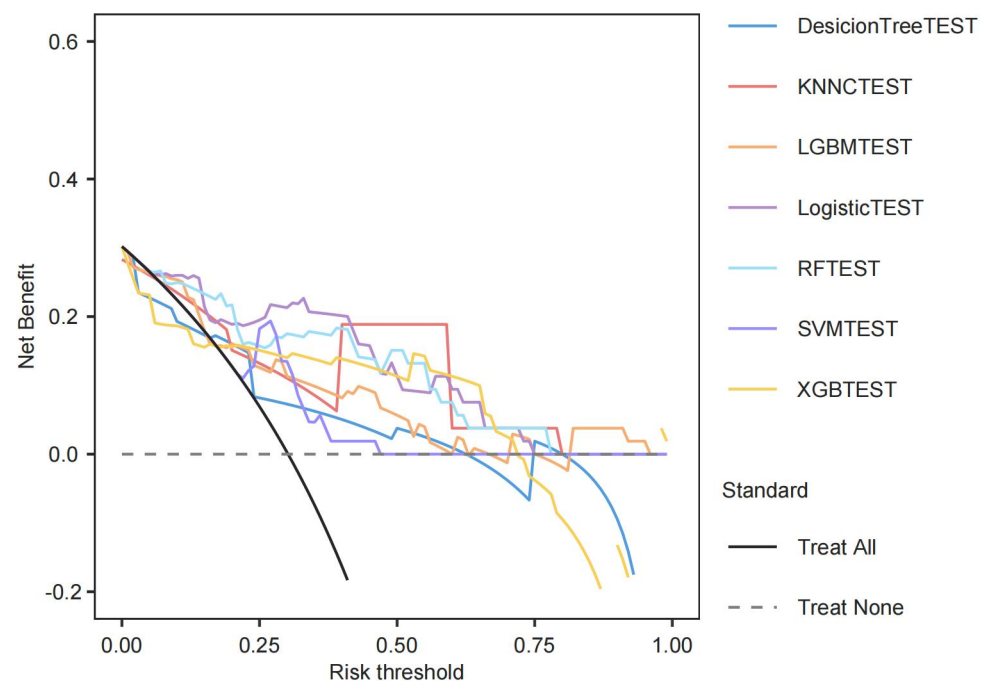**C**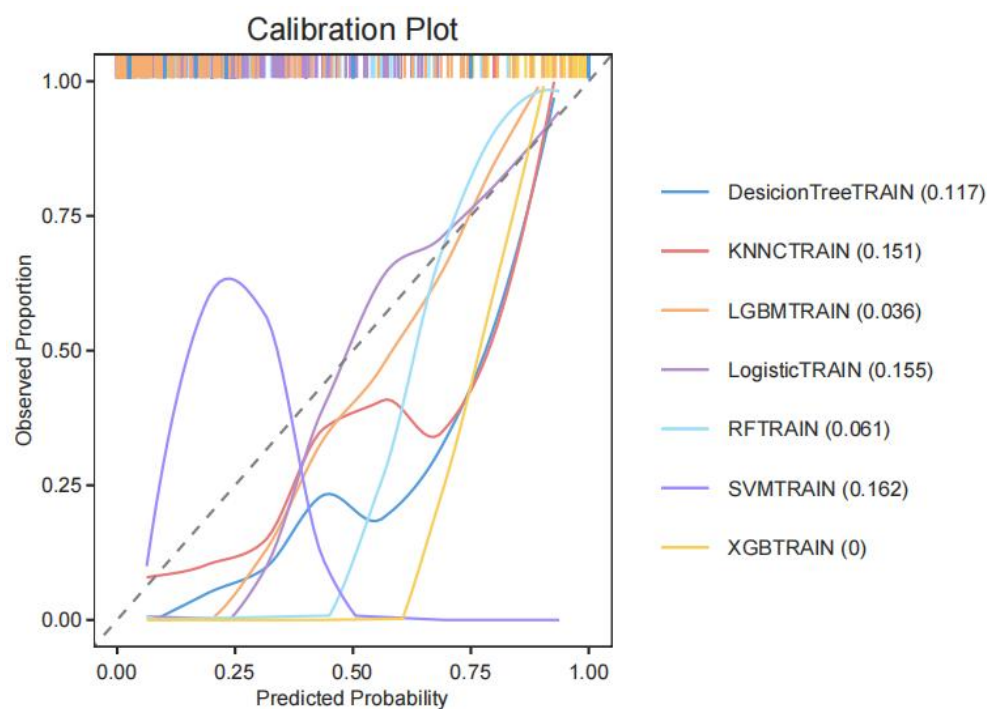**D**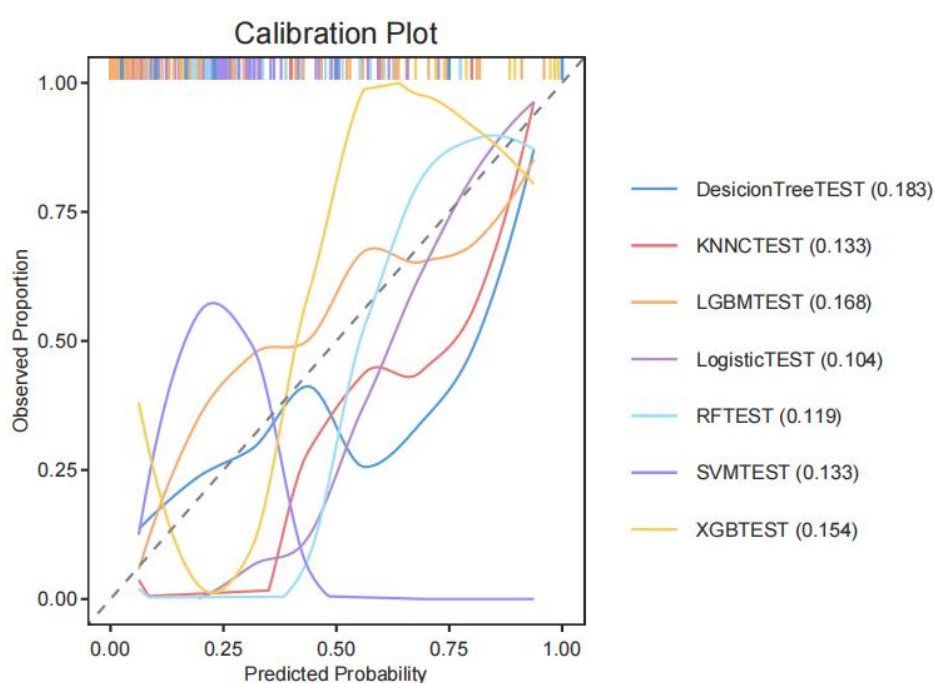**E**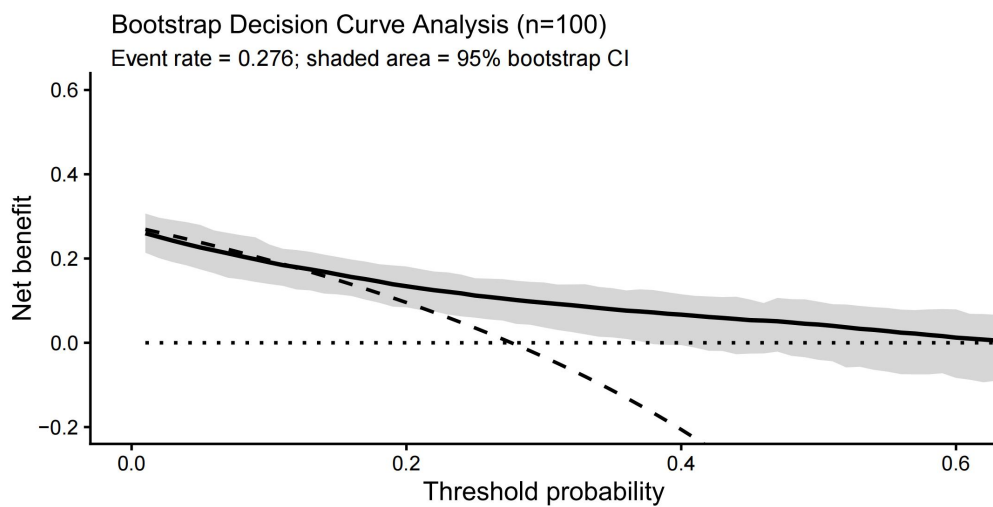**F**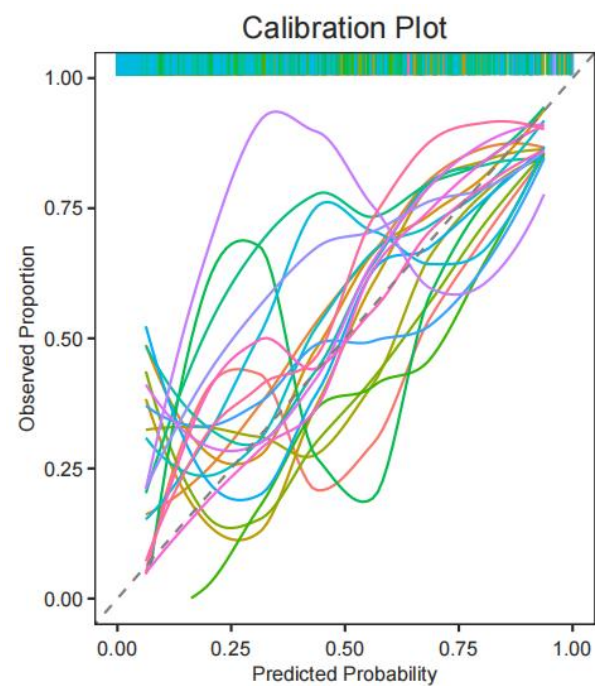

Supplement: Supplementary file 1 [file diagnostics-16-00665-s001.zip › Figure S3.pdf]

**A**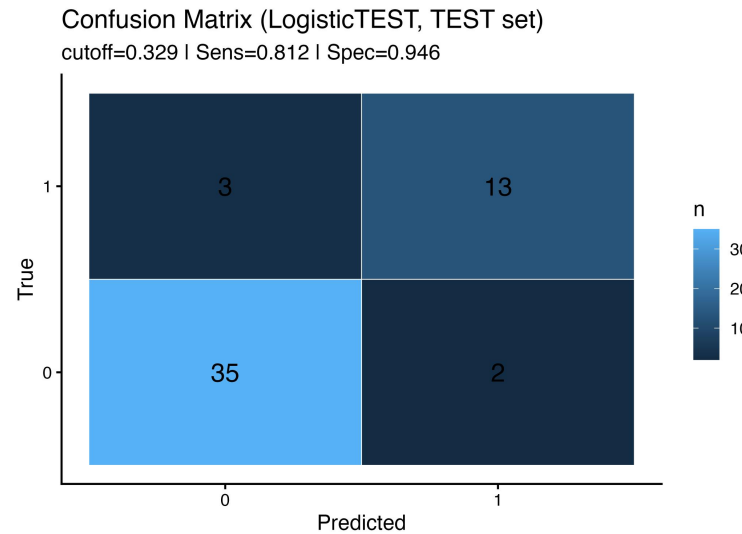**B**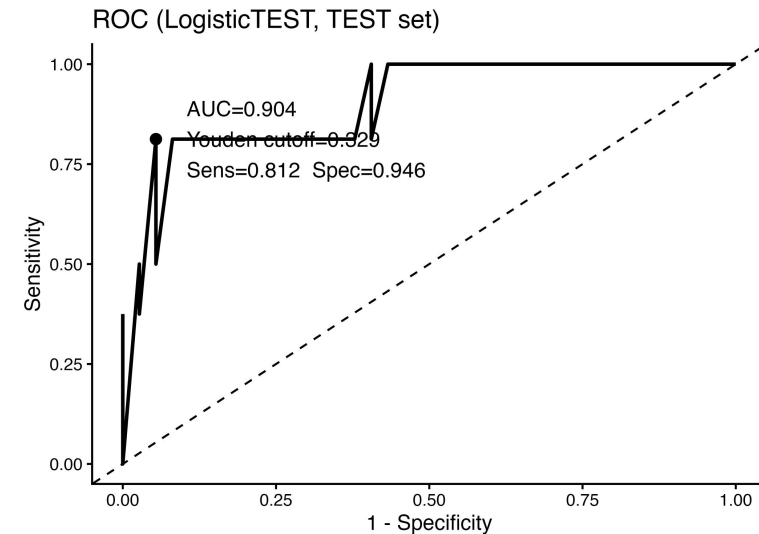**C**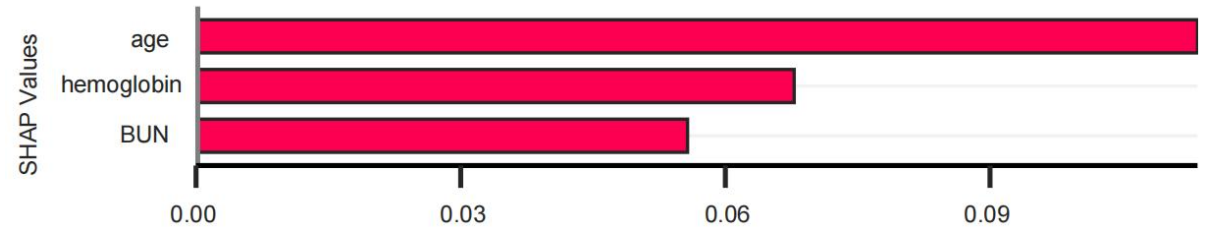

Supplement: Supplementary file 1 [file diagnostics-16-00665-s001.zip › Figure S4.pdf]
